# The Summits of Medical Progress

**Published:** 1894-07-14

**Authors:** 


### The Hospital

An Institutional Journal of

The Medical Sciences and Hospital Administration, with

AN EXTRA NURSING SUPPLEMENT.

Vol. XVI.—No. 407.] [July 14, 1894.

### THE SUMMITS OF MEDICAL PROGRESS.

The competent observer who looks down from a sufficient elevation over the whole field of modern medicine sees cause almost everywhere for deep inward satisfaction. It is not that the startling victories of our art are very numerous; but that the conquest of the unknown is everywhere progressive. Physique, habits, antisepsis, and asepsis are the watchwords beneath which the bold medical warrior fights his way to the Utopia of a world triumphant over disease. Medicine does not advance at one point only, but at every point. Her skilled pioneers are innumerable, her enthusiasms are irresistible. Whether we look to those diseases which spring up within us, or to those enemies which attack us from without; whether we consider the wide ranges of prevention or the more limited areas of cure; in any field and every field we behold the same devotion, the same unwearying energy, the same imperative and not-to be resisted advance.

But among innumerable pioneers there are some who seem to push forward before their fellows; who strive to reach summits more conspicuous than all the surrounding regions. Of such pioneers the inoculators, the preventers, the would-be extirpators of whole armies of diseases, root and branch, must ever be objects of the profoundest interest alike to the medical philosopher and to the man whose proudest distinction it is to be known by the title of "humanitarian."

It is something to encourage the too long neglected doctor to hold his head high to know that a new conquest of India is being attempted in his name: in his name and with his weapons of reasonableness and peace. Last year Dr. Haffkine went forth from Europe on a most momentous errand. Not yet do men sound trumpets when the pioneers of medical science set out on their beneficent enterprises. As yet such demonstrations are reserved for the warrior or the geographer. But, doubtless, as humane science becomes more known and loved, her warriors will not lack the inspiring blast of the trumpet and the sympathetic "God-speed" of the multitudes, which is one of the most powerful of all incentives to heroism and success. The mere mention of medical science in the glowing language which has hitherto been held to be fittest for the warrior is considered by many to be inappropriate. Grey and colourless words and the coldest of phrases are thought to be the russet clothing which best beseems so modest and quiet a spirit of the times. But what if a new conquest of India should be made? What if cholera should be annihilated throughout those dread regions where for centuries uncounted it has held high carnival? May we not then blow the trumpet of victory? May not even the poets of the coming century tune their lyres to music not heard before? We decline to admit that science, the science of life and health, must always don the russet gown. If we mistake not, the near future will clothe her with the garments of the conqueror and erect statues to her honour on every hill.

Dr. Haffkine went out to British India from the Pasteur Laboratory in 1893. It will be remembered that he made claim to having developed a method of entirely preventing cholera, even during epidemic periods, by means of inoculations with cultures of the *comma bacillus*, the bacillus, that is to say, which Koch several years ago declared to be the efficient cause of cholera, and which, after much keen criticism and manifold investigations by rival scientists, has been accepted in every civilised country as the true *fons et origo mali* of that dread disease. At the time of Dr. Haffkine's departure for India a full account was given in the pages of The Hospital of the *modus operandi* of his prophylaxis. In brief, Dr. Haffkine made cultures of the cholera bacillus on different media. For the purposes of his inoculations he produced two special cultures, of different but known and uniform virulence. With these cultures he made two several inoculations of persons exposed to cholera surroundings at intervals of five days. Certain local and general symptoms were set up by the inoculations, but no serious harm was done to any individual. After a varying but short period the inoculées recovered, and were then considered by the inoculator to be thoroughly protected against cholera.

Fortunately for Europe the epidemic of cholera which had prevailed, and to which Dr. Haffkine had owed much of his inspiration, died down; and there was therefore no opportunity for trying his method of prophylaxis on an extensive scale in any European country. But in certain districts of India, cholera, as is well known, is always present, it is endemic.

To India, therefore, Dr. Haffkine was resolved to go, and the endemic area of Bengal was chosen as the scene of his operations. It is now about a year since this enterprise was initiated. During that time every facility has been given to Dr. Haffkine by the British Government, and several series of the most interesting experiments known to medical science have been performed. No fewer than 25,000 persons, both Europeans and natives, of all ranks, have been inoculated, and some most striking results have been obtained. The results obtained are of a kind which appeal quite as readily to the popular as to the medical mind. One or two examples will show their nature. On March 29th, a fatal case of cholera occurred in the house of one Mungloo Jemadar. The whole family consisted of eighteen persons. On the 31st, eleven of the eighteen were inoculated, leaving seven not inoculated. Of the inoculated eleven not one took cholera; of the non-inoculated seven, four took it, of whom three died. Here was surely a very striking set of circumstances. Another series of experiments was on a larger scale. About the end of March two fatal cases of cholera occurred in a population of about two hundred persons grouped around two tanks in Kattal Bagan Bustee. Of the two hundred as many as one hundred and sixteen were aftewards inoculated. After the inoculations there occurred nine more cases of cholera in the bustee, and of these seven died. But the whole nine cases occurred among the eighty-four non-inoculated persons. Not one of the one hundred and sixteen inoculated was attacked. These are examples of the experiments which are being made by Dr. Haffkine under the critical eyes, not only of Mr. Hankin, the well-known Government bacteriologist, but also of all the experienced European medical men who have seen many years of service in the these very cholera regions of Bengal, to which Dr. Haffkine, before he commenced his present enterprise, had been a complete stranger.

Whatever be the final conclusions at which Dr. Haffkine and his coadjutors arrive, it is clear that from the standpoint of medicine, and still more from that of universal humanity, a series of experiments are being made which are not surpassed in interest by a single one of the most eagerly investigated scientific problems of modern times. It is not merely that so puissant a foe as cholera will be conquered if Dr. Haffkine's faith should be justified by facts, but the axe will be laid to the root of the tree of all bacteriological diseases. In vaccination for small-pox we have, as we think, an almost miraculous example of the success of preventive medicine. But if Haffkine should prove a true physiological prophet we shall leave vaccination, so far as science is concerned, a long way in the rear. Vaccination protects, we know not how. The bacillus of vaccination and of small-pox has never yet been discovered. But Haffkine's virus for protection against cholera is a known virus, scientifically understood, scientifically produced, and, so far, effecting results of the highest degree of scientific certainty and uniformity. Well may the medical profession, and all men whose minds vibrate with the new life of modern science and hope, look forward to the developments of the next few months in India as the mother looks forward to the birth of her firstborn child, or the defender of his country to the issues of the great battle which is to make his country free for ever or for ever enslaved!

